# Deep gene selection method to select genes from microarray datasets for cancer classification

**DOI:** 10.1186/s12859-019-3161-2

**Published:** 2019-11-27

**Authors:** Russul Alanni, Jingyu Hou, Hasseeb Azzawi, Yong Xiang

**Affiliations:** School of Information Technology, Deakin University, Geelong, Victoria Australia

**Keywords:** Gene selection, Microarray, Evolutionary algorithms, Gene expression programming

## Abstract

**Background:**

Microarray datasets consist of complex and high-dimensional samples and genes, and generally the number of samples is much smaller than the number of genes. Due to this data imbalance, gene selection is a demanding task for microarray expression data analysis.

**Results:**

The gene set selected by DGS has shown its superior performances in cancer classification. DGS has a high capability of reducing the number of genes in the original microarray datasets. The experimental comparisons with other representative and state-of-the-art gene selection methods also showed that DGS achieved the best performance in terms of the number of selected genes, classification accuracy, and computational cost.

**Conclusions:**

We provide an efficient gene selection algorithm can select relevant genes which are significantly sensitive to the samples’ classes. With the few discriminative genes and less cost time by the proposed algorithm achieved much high prediction accuracy on several public microarray data, which in turn verifies the efficiency and effectiveness of the proposed gene selection method.

## Background

Studying the correlation between microarray data and diseases such as cancer plays an important role in biomedical applications [1]. Microarray data contains gene expressions extracted from tissues (samples). We can obtain more information about the disease pathology by comparing the gene expressions of the normal tissues with the ones of the diseased tissues [1]. Exploring the difference between the cancerous gene expression in tumor cells and the gene expression in normal tissues can reveal important information from microarray datasets, based on which a number of classification techniques have been used to classify tissues into cancerous / normal or into types/subtypes [2–6]. However, microarray data generally has its own high dimensionality problem, i.e., usually there are thousands of genes/attributes but a few samples in a dataset. Moreover, most of these attributes are irrelevant to the classification problem. Therefore, reducing the attribute dimensionality and meanwhile ensuring that the selected attributes still contain rich and relevant information could address this data imbalance problem, although it remains a big challenge. In addition, small sample set makes the problem much harder to solve because the Machine Learning (ML) algorithms do not have enough space to learn (training examples) and this will increase the risk of over fitting. Moreover, microarray data is known as of highly complicated because most of the attributes (genes) in microarray data are directly or indirectly correlated with each other [7]. Selecting a small relevant attribute subset can solve many problems related to microarray data [8, 9]. By removing irrelevant and redundant attributes, we can reduce the dimensionality of the data, simplify the learning model, speed up the learning process and increase the classification accuracy. Several studies have developed and validated a novel gene expression signature and used it as a biomarker to predict cancer in clinical trials [10, 11]. Cancer-associated microarray biomarkers allow less-invasive monitoring and can facilitate patient diagnosis, prognosis, monitoring, and treatment in the oncology field [12, 13].

Several gene selection methods have been developed to select the genes that are directly related to the disease diagnosis, prognosis, and therapeutic targets [14]. In addition to statistical methods, recently data mining and machine learning solutions have been widely used in genomic data analysis [9, 15]. However, still most of the existing gene selection approaches are suffering from several problems such as the stagnation in local optima and the high computational cost [16–18]. Therefore, to solve these problems an efficient new selection approach is needed.

Evolutionary Algorithms (EA) have recently played an important role in gene selection field due to their ability in global search [19]. Besides, many hybrid EA have been proposed to improve the accuracy of the classification methods [20–23]. Various evolutionary algorithms aim to find an optimal sub-set of features by using bio-inspired solutions (such as Genetic Algorithm (GA) [24], Genetic programming (GP) [25], particle swarm optimization (PSO) [26], and Honey Bee [27]). These kinds of algorithms have shown appropriate performances over various problems but are dependent on expert’s intervention to obtain the desired performance.

Recently, a new gene selection method called Gene Selection Programming (GSP) [28] was proposed which showed good results in terms of accuracy, the number of selected genes and time cost. However, the problem of search space is still unsolved.

Gene Expression Programming (GEP) [29] is a new evolutionary algorithm, which was widely used for classification and gene selection [30–35]. GEP has two merits: flexibility which makes it easy to implement, and the capability of getting the best solution, which is inspired by the ideas of genotype and phenotype. In this paper, we use GEP to construct our algorithm.

The purpose (and contribution) of this paper is to present a simple and thus computational efficient algorithm to solve the problem of attribute selection from microarray gene expression data. To this end we explore how to extract the important features from massive datasets.

The rest of this paper is organized as follows: In Gene Expression Program a brief background of GEP is presented. The proposed gene selection algorithm DGS is presented in Results. Evaluation results and discussions, as well as statistical analysis, are presented in Discussion. Finally, Conclusion gives the conclusions.

## Gene expression program

Gene Expression Program (GEP) [36] is an evolution algorithm that creates a computer programing/ model from two parts. The first part, which is also known as genotype, is the characteristic linear chromosomes with a fixed length. Each chromosome consists of one or more genes and each gene consists of a head (h) and a tail (t). The head consists of terminals (attributes) and functions while the tail consists of attributes only, and the head length and tail length follow the rule t = h (n-1) + 1 where n is the maximum number of parameters required in the used functions. The second part is the expression tree (ET) which is also known as phenotype. For example, suppose h = 5 and the chromosome has only one gene. The function set is {+, Q, /} where Q is the square root and the terminals set (the attributes in the data) is coded as {a_0_,…, a_6_} then an example of chromosome could be.

**+/a**_**4**_**Qa**_**2**_a_1_a_5_a_6_a_3_ a_0_ a_3_,(Genotype)

where the bold part represents the head and the rest represents the tail. The ET is.

(Phenotype)

The basic GEP algorithm consists of four steps: creating the chromosomes to initialise the population, evaluating the fitness of each individual/ chromosome by using a predefined fitness function, identifying a suitable stop condition/s and applying the genetic operations to modify the individuals for the next generation. GEP was successfully applied on microarray data to find different biological characteristics [30, 37]. More details about GEP algorithm and process can be found in [29, 36, 38].

## Results

### Materials

In our experiments, we evaluated the performance of DGS method on an integrated lung cancer microarray dataset downloaded from NCBI (https://www.ncbi.nlm.nih.gov/geo/query/ acc.cgi?acc=GSE68465). The dataset contains 442 patients collected from 4 hospitals: Moffitt Cancer Center (MCC) 79 patients, Memorial Sloan-Kettering Cancer Center (MSKCC) 104 patients, University of Michigan Cancer Center (UMCC) 177 patients, and Dana Farber Cancer Centre (DFCC) 82 patients.

The data include various prognosis information, we used lung cancer recurrence information to predict the lung cancer recurrence. To this end, we extracted only the samples with recurrence or free survival (non-recurrence) and delete all the unrelated information such as the dead patients and the disease-free patients. After the preparation the total number of the patients in the dataset was 362. The number of cancer recurrence patients was 205 while the number of free survival patients was 157. The total number of attributes (probe sets) was 22,283. Regarding the training and testing of the method, we used 10-fold cross- validation method. The 9 folds were used for training DGS while the left fold was used for testing. For more reliability we repeated the experiment ten times and obtained the average results of these experiments.

To make the evaluations more reliable, we validated the prediction model using another independent dataset with the same statistical measures. The validation dataset from South Korea (GSE8894) can be downloaded from NCBI. GSE8894 dataset had 138 NSCLC samples from Affymetrix Hu133-plus2 platform microarray chips. It had an equal number of samples for two classes, i.e. 69 samples were labelled ‘recurrence’ and 69 samples were labelled ‘nonrecurrence’.

### The best setting for the number of chromosome (CH) and the number of genes (N)

To find out the best settings for the number of chromosomes in each generation (CH) and the number of genes (N) in each chromosome, we did experiments with different values of CH and N. To show the effect of CH and N on the DGS classification performance, we selected nine different settings. Three different values for CH, 100, 200 and 300, and for each CH value, three different N values are selected: 1, 2 and 3. The values of CH are increased by 100 to make the effect of CH values clear, especially when the effect of increasing CH is very slight. To make the experiments more reliable, we repeated the experiment 10 times and took the average as a final result. The parameters used in DGS, which is based on gene expression programming (GEP) algorithm, are showed in Table 1.

**Table 1:** Parameters used in DGS

Parameter	Setting
Terminal set	Start with all the attributes in microarray dataset.
Function set	+, −, ÷, Q where Q is the square root
Maximum Iterations number	200
Mutation	0.044
Recombination	0.3

The average experimental results are presented in Table 2. *AC*_*avg*_, *I*_*avg*_*, S*_*avg*_ and *TM*_*avg*_ represent the average accuracy, the number of iterations, the number of selected attributes and CPU time respectively for ten runs, while *AC*_std_, *I*_*std*_, *S*_std_. and *TM*_std._ represent the standard deviation of the classification accuracy, the number of iterations, the number of selected attributes and CPU time respectively.

**Table 2:** the results of different setting for the number of genes (N) and the number of chromosomes (CH)

genes(N)	CH	AC avg.	I avg	S avg.	TM avg.
1	100	77.92	200	7.37	189.00
	200	85.45	192.50	10.07	247.28
	300	86.18	152.40	4.00	285.01
	average	83.18	181.63	7.15	240.43
2	100	82.29	191.30	4.00	183.52
	200	87.49	145.90	3.90	218.85
	300	87.54	144.03	3.90	279.74
	average	85.77	160.41	3.93	227.37
3	100	87.20	144.00	3.90	204.72
	200	87.54	135.00	3.90	288.05
	300	87.54	135.00	3.90	362.05
	average	87.43	138.00	3.90	284.94

We observed from Table 2 that:

1. Comparing CH with N: CH has a less effect on the results than N.
2. Regarding CH results: CH has positive relationships with *AC*_*avg*_*, TM*_*avg*_ *and S*_*avg.*_That is when CH value was increased, *AC*_*avg*_*, TM*_*avg*_ *and S*_*avg.*_ values also increased. While CH has negative relationships with *AC*_*std*_*, TM*_*std.*_ *and S*_*std*_. That is when CH values increased, *AC*_*std*_*, TM*_*std.*_ *and S*_*std*_. values were decreased. The results became stable when the CH was over 200.
3. Regarding N results: N has positive relationships with, AC_avg_*, TM*_*avg*_ and *S*_*avg*_ and negative relationships with *AC*_*std*_*, TM*_*std.*_ and *S*_*std*_. The results became stable after two genes.
4. Increasing CH values over 200 would increase the processing time while the *AC* and *N* results would not significantly change.
5. The best results were achieved when the value of CH is 200 and the value of N is 2.

### DGS evaluations

#### Evaluate DGS performance based on the AC, SN, SP, PPV, NPV, S, TM and AUC

The performance of DGS was evaluated and measured for each test in terms of classification accuracy (AC), Sensitivity (SN), Specificity (SP), Positive predictive value (PPV), Negative predictive value (NPV), the number of selected genes (S), and processing time (TM) with confidence intervals (CI 95%).

To make the evaluations more reliable, we compared DGS with five representative models on the integrated lung cancer dataset. These five gene selection algorithms were Correlation-based Feature Selection (CFS), Consistency Subset Feature Selection (CSFS), Wrapper Subset (WS), Support Vector Machine (SVM) which applied using WEKA with their default configurations, and Gene Expression Programming (GEP) using GEP4J package. All the values are the average (avg) values over ten runs of the models. Table 3 gives the performance evaluation values for all the prediction models.

**Table 3:** Comparison of DGS performance with different feature selection models in term of AC, SN, SP, PPV, NPV, AUC, S and TM with CI 95% for each test

	CSF	CSFS	WS	SVM	GEP	DGS
AC _avg_.	0. 8436	0.8370	0.8395	0.8544	0.8577	0. 8749
CI 95%	±0.1921	±0.1279	±0.1180	±0.0986	±0.0922	± 0.1287
SN _avg_.	0.8995	0.8907	0.8932	0.9307	0.9278	0.9522
CI 95%	±0.2520	±0.1893	±0.1753	±0.1362	±0.1575	±0.1187
SP _avg_	0.7707	0.7669	0.7694	0.7548	0.7662	0.7739
CI 95%	±0.5809	±0.3157	±0.3417	±0.1682	±0.1001	±0.2569
PPV _avg_.	0.8373	0.8332	0.8351	0.8321	0.8382	0.8462
CI 95%	±0.2956	±0.1652	±0.1744	±0.0910	±0.0637	±0.1362
NPV_avg_.	0.8550	0.8434	0.8468	0.8931	0.8907	0.9253
CI 95%	±0.3803	±0.2855	±0.2557	±0.2475	±0.2749	±0.2401
AUC_avg_.	0.8293	0.8104	0.8414	0.8499	0.8423	0.8687
CI 95%	±0.0223	±0.0213	±0.0211	±0.0218	±0.0216	±0.0210
S_avg._	6.5	6.9	6.7	6.3	6.2	3.9
CI 95%	±0.8430	±0.978	±1.0013	±1.3016	±0.9917	±0.3338
TM _avg_	600.12	600.02	600.01	600.21	620.51	218.85
CI 95%	±0.1821	±0.0189	±0.0134	±0.3700	±24.6415	±34.6227

In term of AC, the experimental results showed that the DGS method achieved the highest average accuracy result (0. 8749), while the average accuracies of other methods were 0.8436, 0.8370, 0.8395, 0.8544 and 0.8577for CSF, CSFS, WS, SVM and GEP respectively.

In term of SN, the experimental results showed that the DGS method achieved the highest average accuracy result (0. 9522), while the average sensitivity results of other methods were 0.8995, 0.8907, 0.8932, 0.9307and 0.9278 for CSF, CSFS, WS, SVM and GEP respectively.

In term of SP, the experimental results showed that the DGS method achieved the highest average accuracy result (0. 7739), while the average sensitivity results of other methods were 0.7707, 0.7669, 0.7694, 0.7548 and 0.766242 for CSF, CSFS, WS, SVM and GEP respectively.

The DGS model achieved the highest average PPV which was 0. 8462, while the average PPV of other models were 0.8373, 0.8332, 0.8351, 0.832 and 0.8382 for CSF, CSFS, WS, SVM, GEP respectively.

The highest average NPV was for DGS (0. 9253) while the average PPV of other models were 0.8550, 0.8434, 0.8468, 0.8931 and 0.8907 for CSF, CSFS, WS, SVM, GEP respectively.

DGS achieves the smallest number of selected genes (3.9) which is almost half of the number of genes selected by other comparison methods.

Regarding TM, the less processing time was for DGS (218.85) while the average time results of other models were 600.12, 600.02, 600.01, 600.21 and 620.51 for CSF, CSFS, WS, SVM, GEP respectively.

**Fig. 1:**
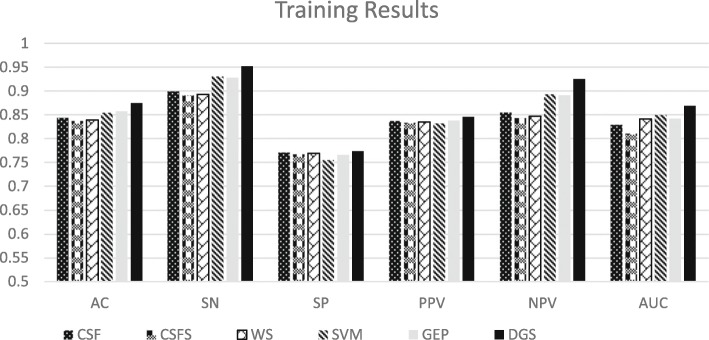
Comparison of DGS performance with different feature selection models in term of AC, SN, SP, PPV, NPV and AUC

Figure 1 shows the effectiveness of DGS method in term of AC, SN, SP, PPV, NPV, S, TM and AUC.

For more reliability, we validated the prediction model using an independent dataset (GSE8894). The selected genes were used as biomarkers to classify the recurrence/ non-recurrence patients. The evaluation results for DGS on the validation dataset in terms of AC, SN, SP, PPV, NPV and AUC are presented in Table 4, which show the effectiveness of the proposed gene selection algorithm DGS that enabled the prediction model to achieve the accuracy of 87.68%.

**Table 4:** Validation results of DGS on the independent dataset GSE8894

AC _avg_.	0.8768	PPV _avg_.	0.8714
CI 95%	±0.1932	CI 95%	±0.5191
SN _avg_.	0.8841	NPV_avg_.	0.8824
CI 95%	±0.2360	CI 95%	± 0.3148
SP _avg_	0.8696	AUC_avg_.	0.8686
CI 95%	±0.4721	CI 95%	±0.0210

**Fig. 2:**
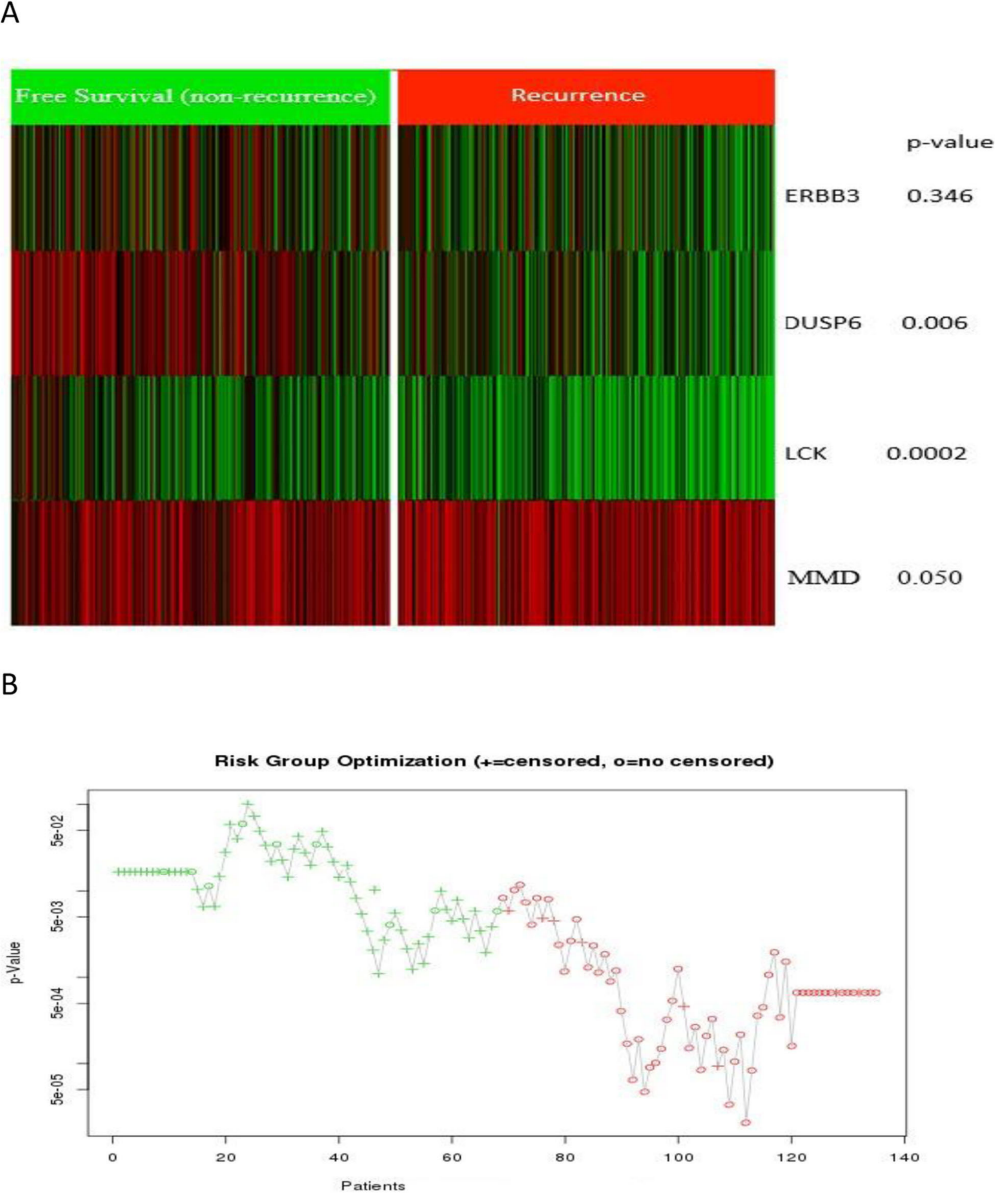
The evaluation results for the selected genes. **a**The gene expression level of the selected genes shown as a heatmap. **b** The prediction results using the selected genes

Figure 2 shows that the selected genes are able to separate risk groups (recurrence/non-recurrence) characterized by differences in their gene expressions.

### The biological meaning for the selected genes from DGS method

In this section we present the biological meanings of the selected genes obtained from “Expression Atlas” database of EMBL-EBI (http://www.ebi.ac.uk/gxa/). Table 5 shows the genes that were selected by DGS method for the ten runs.

**Table 5:** The selected gens of each run

Run number	S	Probe ID	Gene symbol
1	4	204891_s_at	LCK
		208893_s_at	DUSP6
		202454_s_at	ERBB3
		202885_s_at	MMD
2	4	204891_s_at	LCK
		208893_s_at	DUSP6
		202454_s_at	ERBB3
		202885_s_at	MMD
3	4	204891_s_at	LCK
		208893_s_at	DUSP6
		202454_s_at	ERBB3
		202885_s_at	MMD
4	4	204891_s_at	LCK
		208893_s_at	DUSP6
		202454_s_at	ERBB3
		202885_s_at	MMD
5	4	204891_s_at	LCK
		208893_s_at	DUSP6
		202454_s_at	ERBB3
		202885_s_at	MMD
6	3	204891_s_at	LCK
		208893_s_at	DUSP6
		202454_s_at	ERBB3
7	4	204891_s_at	LCK
		208893_s_at	DUSP6
		202454_s_at	ERBB3
		202885_s_at	MMD
8	3	208893_s_at	DUSP6
		202454_s_at	ERBB3
		202885_s_at	MMD
9	4	204891_s_at	LCK
		208893_s_at	DUSP6
		202454_s_at	ERBB3
		202885_s_at	MMD
10	5	204891_s_at	LCK
		208893_s_at	DUSP6
		202454_s_at	ERBB3
		202885_s_at	MMD
		205027_s_at	MAP3K8

We used the OMIM, Expression Atlas and NCBI websites to find the biological meanings of the selected microarray probe-ids and list their corresponding genes. The specifications are shown in Table 6.

**Table 6:** The final selected genes from the gene selection method DGS

Gene symbol	Gene Name	Chr.	NCBI UniGene number	Specification
LCK	lymphocyte-specific protein tyrosine kinase	1	3932	The encoded protein is a key signaling molecule in the selection and maturation of developing T-cells
DUSP6	dual-specificity phosphatase6	12	1848	This gene inactivates (ERK2), resulting in tumor suppression and apoptosis. The protein encoded by this gene is a member of the dual specificity protein phosphatase subfamily
ERBB3	v-erb-b2 avian erythroblastic leukemia viral oncogene homolog 3	12	2065	Also known as HER3 (human epidermal growth factor receptor 3) This gene encodes a member of the epidermal growth factor receptor (EGFR) family of receptor tyrosine kinases which are often aberrantly expressed and/or activated in human cancers
MMD	monocyte-to-macrophage differentiation associated protein	17	23,531	This protein is expressed in mature macrophages but the function of this protein is still unknown.

Note: NCBI UniGene number with more information about the genes can be found from NCBI website https://www.ncbi.nlm.nih.gov/geo/

### DGS comparison with up-to-date models

We also compared DGS method with models recently proposed, which are IBPSO [39], IG-GA [40], IG-ISSO [41], EPSO [42], mABC [43] and IG-GEP [32]. The comparison results were based on two criteria: the classification accuracy and the number of the selected genes regardless of the methods of data processing.

We used the same datasets that were used by these up-to-date models to compare DGS results. A brief description of these datasets is presented in Table 7.

**Table 7:** Description of the experimental datasets

No.	Dataset	Samples(X)	Number of Genes(Y)	Classes	Reference
1	11_Tumors	174	12,533	11	[44]
2	9_Tumors	60	5726	9	[45]
3	Brain_Tumor1	90	5920	5	[46]
4	Brain_Tumor2	50	10,367	4	[47]
5	Leukemia 1	72	5327	3	[48]
6	Leukemia 2	72	11,225	3	[49]
7	Lung_Cancer	203	12,600	5	[50]
8	SRBCT	82	2308	4	[51]
9	Prostate_Tumor	102	10,509	2	[52]
10	DLBCL	77	5469	2	[53]

The comparison results are presented in Table 8. Across the ten datasets used in the comparison, DGS achieved the best results in seven datasets (11_Tumors, 9_Tumors, Leukemia1, Leukemia2, Lung_ Cancer, DLBCL and SRBCT) compared with the other comparator models, while mABC achieved better results in three data sets (Prostate, Brain_Tumor1, and Brain_Tumor2). Moreover, DGS achieved superior results in term of the number of selected genes which were the best results in all experimental datasets. The average evaluation values in terms of accuracy (AC_avg_) and the number of selected genes (S_avg_) for IBPSO, IG-GA, IG-ISSO, EPSO, mABC and IG-GEP are listed in Table 8.

**Table 8:** Comparison of the gene selection algorithms on ten selected datasets

11_Tumors	IBPSO	IG-GA	IG-ISSO	EPSO	mABC	IG-GEP	DGS
AC _avg._	95.06	92.53	95.92	95.4	99.5	93.88	99.88
AC _std._	0.3	_____	1.31	0.61	0	3	0.01
S _avg._	240.9	479	19.8	237.7	47.27	18.6	17.9
S _std._	9.55	____	2.57	9.66	7.79	3	1.2
9_Tumors	IBPSO	IG-GA	IG-ISSO	EPSO	mABC	IG-GEP	DGS
AC avg.	75.5	85	91.67	75	98.65	89.83	98.89
AC _std._	1.58	____	2.48	1.11	0.01	1.01	0.02
S _avg._	240	52	15.7	247.1	34.73	20.3	13.7
S _std._	7.95	____	2.2136	9.65	5.54	2.1	1.02
Brain_Tumor1	IBPSO	IG-GA	IG-ISSO	EPSO	mABC	IG-GEP	DGS
AC _avg._	92.56	93.33	98	92.11	100	96.11	99.82
AC _std._	0.54	____	0.88	0.82	0	1.41	0.31
S _avg._	11.2	244	10.1	7.5	16.87	19	9.2
S _std._	7.15	____	1.73	2.51	2.85	1.05	1.5
Brain_Tumor2	IBPSO	IG-GA	IG-ISSO	EPSO	mABC	IG-GEP	DGS
AC _avg._	91	88	99.8	92.4	100	99.8	99.9
AC _std._	0.05	____	0.63	1.27	0	1.01	0.1
S _avg._	6.4	489	10.4	6	10.52	14.6	9.8
S _std._	1.9	____	1.08	1.83	1.72	0.7	0.4
Lung_ Cancer	IBPSO	IG-GA	IG-ISSO	EPSO	mABC	IG-GEP	DGS
AC _avg._	95.86	95.57	99.41	95.67	100	98.48	100.00
AC _std._	0.53	____	0.45	8.3	0	0.61	0.00
S _avg._	14.9	2101	10.4	8.5	23.31	14.5	8.30
S _std._	10.57	____	1.08	2.11	5.14	0.61	0.82
Leukemia1	IBPSO	IG-GA	IG-ISSO	EPSO	mABC	IG-GEP	DGS
AC _avg._	100	100	100	100	100	100	100
AC _std._	0	____	0	0	0	0	0
S _avg._	3.5	82	4.6	3.2	5.67	7.7	2.9
S _std._	0.71	____	0.52	0.63	0.73	0.67	0.63
Leukemia2	IBPSO	IG-GA	IG-ISSO	EPSO	mABC	IG-GEP	DGS
AC _avg._	100	98.61	100	100	100	100	100
AC _std._	0	____	0	0	0	0	0
S _avg._	6.7	782	4.2	6.8	6.29	7.5	4.1
S_std._	1.5	____	0.42	2.2	0.98	1.58	0.73
SRBCT	IBPSO	IG-GA	IG-ISSO	EPSO	mABC	IG-GEP	DGS
AC _avg._	100	100	100	99.64	100	______	100
AC _std._	0	____	0	0.58	0	_______	0
S _avg._	17.5	56	4.3	14.9	5.59	_____	4
S _std._	8.32	____	0.48	13.03	0.51	______	0.67
Prostate	IBPSO	IG-GA	IG-ISSO	EPSO	mABC	IG-GEP	DGS
AC _avg._	97.94	96	98.82	97	100	98.33	99.87
AC _std._	0.31	____	0.41	0.62	0	0.4	0.52
S _avg._	13.6	343	8.4	6.6	10.73	18.1	8.2
S_std._	7.68	____	1.78	2.17	3.15	0.9	0.79
DLBCL	IBPSO	IG-GA	IG-ISSO	EPSO	mABC	IG-GEP	DGS
AC _avg._	100	100	100	100	100	______	100
AC _std._	0	____	0	0	0	____	0
S _avg._	6	107	3.9	4.7	4.05	____	3.5
S _std._	1.25	____	0.32	0.82	0.78	____	0.5

## Discussion

We improve the genetic operations that can improve the generation quality effectively. The experimental results show that the proposed DGS can provide a small set of reliable genes and achieve higher classification accuracies in less processing time.

These superior achievements are due to the following DGS features -

1. The ability of DGS to reduce the complexity by using different ways
  - Narrowing the search space gradually. In each iteration DGS extract a new terminal set by removing the genes that don’t provide high fitness values (see DGS Population Generation)
  - Reducing the generation size by applying Eq. 3. (see Generation size controlling)
2. The ability to select the related genes. In each generation DGS removes the unrelated genes to increase the probability of choosing related genes for generating 200 chromosomes, and after several generations DGS can finally find the most related genes. Table 5 shows the gene selection process and results.
3. DGS is faster compared with other comparative methods. This feature comes from the DGS’s abilities.

- The ability of narrowing the search space.
- The ability of resizing the chromosomes in each iteration

Table 9 shows the differences between DGS and the related methods GA and GEP.

**Table 9:** the differences between DGS, GA and GEP

	DGS	GA	GEP
number of chromosomes in each generation	Same number	Same number	Same number
Chromosome length	Flexible length	Fixed length	Flexible length
Generation size	changeable size	Fixed size	Fixed size
Genetic Operation	Systematic selection	Random selection	Random selection
Terminal set	Different set in each generation	Same set in each generation	Same set in each generation

## Conclusion

In this paper, an innovative DGS algorithm is proposed for selecting informative and relevant genes from microarray data sets to improve cancer classifications. The proposed method inherits the evolutionary process from GEP. DGS has the ability of reducing the size of attribute space iteratively and achieve the optimal solution. We applied this method on an integrated dataset and selected 4 genes which can achieve better classification results.

## Method

### Proposed method

**Fig. 3:**
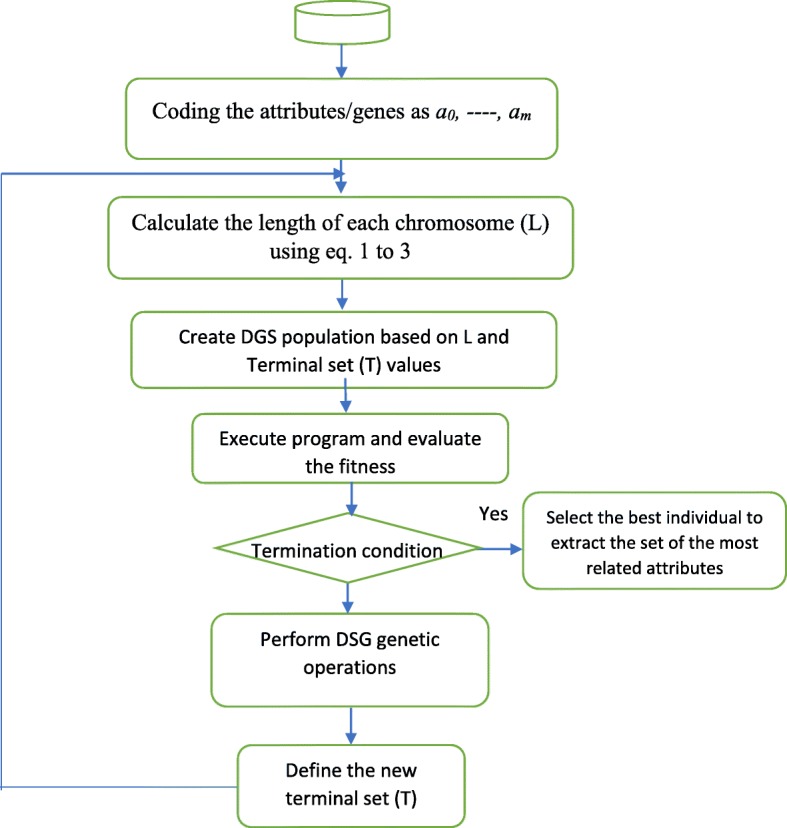
DGS Flowchart

A novel evolutionary method named Deep Gene Selection (DGS) is presented in this section, which is based on the gene expression programming (GEP) algorithm. DGS is developed to explore the subset of highly relevant genes. The proposed evolutionary method consists of several steps as depicted in Fig. 3. According to Fig. 3, the attributes/genes are coded as a_0_, ----, a_m_ where m represents the number of attributes in the dataset. T is the size of the terminal set which is used to create a population of chromosomes. In the first-generation T = m.

The length of each chromosome (L) is defined based on the dimensionality of the dataset. Furthermore, the minimum length of L could also be defined. Next, the population is evaluated using a fitness function that employs a classifier and the number of the attributes. After being assigned fitness values, all chromosomes of the population are sorted to find the best individuals that have the higher fitness values. Improved genetic operators are then applied to the selected population individuals and accordingly the top individuals (the individuals with the highest fitness values) are selected to generate the next generation. Then a new attribute subset with new T is extracted from these best individuals of the new generation. In other words, the output (new attribute set) of previous generation is the input of the next generation. After several generations, the attribute set will represent the minimum genes that can achieve the highest fitness values, because in each generation only the attributes that can achieve the highest fitness values will be selected. One termination condition of this iteration process is that there is no change in the top fitness values. This means the selected genes are the same (same attribute set) and the classification results are the same. Another termination condition is the number of generations reaches the maximum number although the program cannot reach the ideal solution. The selection operation will stop once one of these two termination conditions is met. The application of this algorithm on real data sets is presented in Materials. It is worth noting that the proposed method is taking the advantages of evaluation algorithms and dynamic attribute extraction to reach the optimal solution in a very simple and effective way.

Overall, the proposed method focuses on searching for superior solutions with the smallest number of attributes by using the evolutionary structures to evaluate the best solution and using the dynamic attribute extraction approach to narrow the search space. With the progress of iteration, the cost of search will decrease, and the quality of the solution will increase until the optimal solution (or the solution close to the optimal one) in the smallest space is achieved. DGS was implemented using Java. To implement the expression tree (ET), we used GEP4J package [54]. The DGS flowchart is presented in Fig. 3.

The detailed descriptions of the proposed method, including chromosome representation, initial DGS population, DGS fitness function and improved genetic operations, are presented in the following sub-sections.

### DGS population generation

DGS population is the base of the proposed method. The chromosome concept and representation of DGS population are inherited from gene expression programming (GEP) algorithm (see section 2.2). The chromosomes are constructed from two sets: terminal set (ts) and function set (fs). The function set can be a set of any mathematic operators such as {−, +, /, *, sqr, log}. Terminal set in this paper represents the attribute set of the microarray dataset.

The first generation is generated from all attributes in the microarray dataset. Each individual (chromosome) of the generation is evaluated by the fitness function and assigned a fitness value. All the individuals are then sorted in a descending order from the highest individuals (the individual with the highest fitness value) to the lowest individual. Then the attributes of the first 50% individuals are extracted to generate a new terminal set (ts) for generating the next generation. This means the attribute output of an iteration will be the input of the next iteration for generating a new generation. This iterative population generation process will continue until one of the program termination conditions is met. In this way, DGS is able to reduce the dimension of the attribute search space by extracting the attributes that can achieve the high fitness values.

The details of this population generation process are outlined in Algorithm.1.

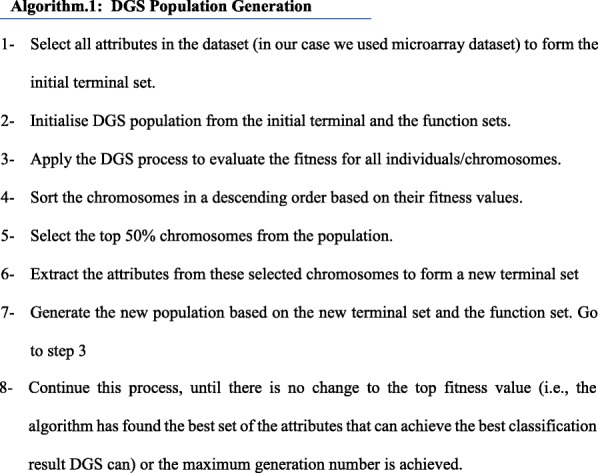



The following simulation example illustrates the generation of a DGS population.

#### Example 1

If we have a dataset that has13 attributes, then.

ts = {a_1_, a_2_, a_3_, a_4_, a_5_, a_6_, a_7_, a_8_, a_9,_ a_10_, a_11_, a_12_, a_13_}.

Let h = 3 and fs = {+. -, *, /, Q} then *n* = 2, t = h (n-1) + 1 = 4 and the gene length g = h + t = 7. Suppose each chromosome has only one gene. The population with 10 individuals/chromosomes, as well as their fitness values, is listed below:

Take chromosome 0 as an example to show how to calculate the fitness function.

+,-,a12 is the head, and a9,a3,a11 , a7 is the tail of chromosome 0.

The Phenotype/ET of chromosome 0 is.

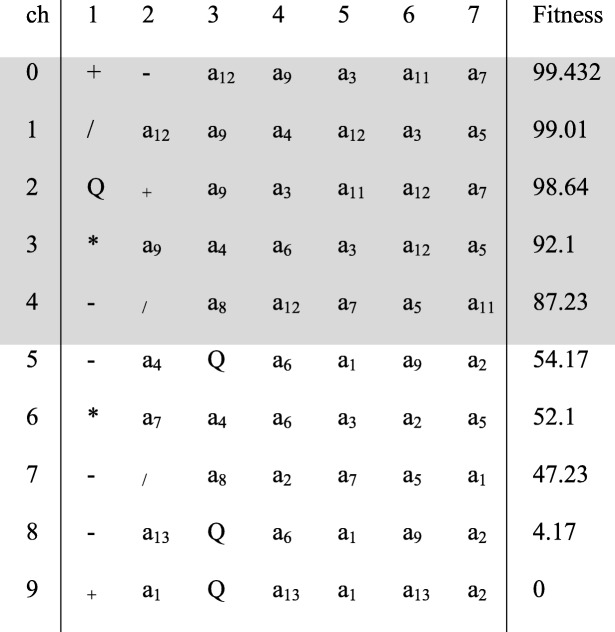

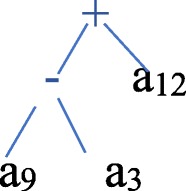





DGS will use the gene expression of a_12_, a_9_, a_3_ genes to calculate the fitness.

DGS sorts the individuals in a descending order based on their fitness values, then selects the top 50% individuals from them (the highlighted individuals in the above example). DGS then extracts the attributes from these selected individuals to form a new terminal set which is {a3, a4, a5, a6, a7, a8, a9, a11, a12}.

DGS will use this new terminal set which is smaller than the original one and the function set to generate a new population. This process will continue until the program reaches the best solution (e.g., Accuracy = 100%) with no changes to the consecutive terminal sets, or the program reaches the maximum number of generations.

### Generation size controlling

The generation size is determined by three values: the number of individuals/ chromosomes (CH) in a generation, the length of each chromosome (L) and the size of the terminal set (T). The generation size must be properly defined. If the size is too big, it will lead to the increment of the computational time, and if it’s too small, the generation may not cover all attributes /terminals. In the original evolution algorithms, the number of chromosomes in each generation (i.e., the generation size) is fixed, so the other values that are suitable for the first generation, are also suitable for all other generations. However, in our method, the first generation is generated from all attributes, and the number of attributes may be thousands in the big datasets. The attributes used for generating the second generation are a subset of the attributes of the first generation as we see in example 1. Usually, the number of attributes used for generating a generation is dynamic, i.e. it decreases or non-decreases with the progress of the evolution program. Therefore, the values of CH and L that are suitable for a generation may not be suitable for other generations. To ensure the generation size is properly defined, we define the following rule in Eq. (1) for these three values.

1 $$ L\ast CH= 2T $$

Actually L*CH is the overall size of a generation in terms attributes and functions. The constant 2 in Eq. (1) is to ensure that each attribute in the terminal set has nearly a double chance to be selected to generate a generation.

Our previous experiments [32] showed that the value of L has more impact on classification results and computational time than CH. So usually we use a fixed CH value (200) for all generations and changeable values for L.

In fact, let N be the number of genes of a chromosome/individual, then

$$ \mathrm{L}=\mathrm{N}\ast \left(\mathrm{gene}\ \mathrm{length}\right)=\mathrm{N}\ast \left(\mathrm{h}+\mathrm{t}\right) $$

where h is the length of gene head and t is the length of gene tail, and

2 $$ t=h\ast \left(n-1\right)+1 $$

where n represents the maximum number of parameters needed in the function set.

From our experiments, we found that *N* = 2 can provide the best classification results from microarray data sets. If we choose N = 2, then

$$ L=2\left(n\ast h+1\right) $$

Considering Eq. (1), we have

$$ 2\left(n\ast h+1\right)\ast CH=2T $$

$$ h=\left(T/ CH-1\right)/n $$

Usually *n* = 2 for commonly used functions, therefore h can be defined as the integer number of (T/CH-1)/n, i.e.

$$ h=\mathrm{floor}\left[\left(T/ CH-1\right)/n\ \right] $$

On the other hand, it is necessary to set a minimum value of h (h = 3 which is a commonly used value) to guarantee the genes of a chromosome contain enough information for evolution.

Based on the above rules and the minimum requirement, we can define the head size (h) of each gene in a chromosome as:

3 $$ h=\mathit{\max}\ \left( 3, floor\ \left[\left(T/ CH- 1\right)/ 2\right]\right) $$

Since CH is fixed (e,g. 200) and the number of genes in a chromosome is set as 2, once the value of h is defined according to (3), the overall size of a generation is defined. The following simulation example shows different h values with different sizes (T) of terminal set.

#### Example 2

If a microarray dataset originally has 2200 attributes and we set CH = 150, the values of h and T are listed in Table 10.

**Table 10:** The results of example 2

Generation	T	h	Generation	T	h
1	2200	7	11	650	3
2	2000	6	12	402	3
3	1852	6	13	254	3
4	1723	5	14	102	3
5	1583	5	15	79	3
6	1296	4	16	53	3
7	1101	3	17	31	3
8	972	3	18	19	3
9	801	3	19	**5**	3
10	734	3	20	**5**	3

### Fitness function

The purpose of using gene selection methods is to obtain a smallest gene subset that can provide the best classification results. To this end, a new fitness function is proposed to enable DGS to select the best individuals/chromosomes. The fitness value of an individual *i* can be calculated by the following equation

4 $$ {f}_i=\left(1-r\right)\ast AC(i)+r\ast \frac{t-{s}_i}{t} $$

This fitness function consists of two parts. The first part is based on the classification accuracy AC(i) of the individual i. We use support vector machine (SVM) as a classification method to calculate the accuracy of an individual/chromosome because it is a powerful classification algorithm which is widely used to solve the binary and multi-classification problems [55, 56] and can achieve a high classification accuracy. To calculate the AC, we use the following Eq. (5), which is widely used in cancer classification.

5 $$ AC=\left( TP+ TN\right)/\left( TP+ FN+ TN+ FP\right) $$

where TP, TN, FP and FN represent True Positive, True Negative, False Positive and False Negative respectively. The second part is based on the number of selected genes, specifically t is the total number of attributes in the terminal set and s_i_ is the selected number of attributes in the individual/chromosome *i*, *r*ϵ [0,0.5) is a predefined weight controlling the importance of *AC*(*i*) and s_i_.

### Improved genetic operations and DGS algorithm

The reason of using genetic operations is to improve the individuals for achieving the optimal solution. In this paper, we improve two genetic operations: Mutation and Recombination. The improved genetic operations depend more on the weight of genes, as we explain below.

#### Attribute weight

The weight (*w*) of each attribute (*i*) is calculated based on Eq. (6)

6 $$ {w}_i=\frac{k_i}{sum}\kern0.5em \in \left(0,1\right) $$

where $sum=\sum \limits_i{k}_{i\kern0.5em }\kern4em i\in ts$, *k*_*i*_ is the rank value of the attribute *i*, and $\sum \limits_{i\ }{w}_i=1$ .

In this study we used Gain Ratio to calculate the rank of the individual *i* as follow:

7 $$ {k}_i=\frac{information\ gain\ \left(i\ \right)}{intrinsic\ information\ (i)} $$

The details of calculating the information gain and the intrinsic information can be found in [57–59].

The attributes with a higher weight contain more information for classification.

#### Mutation

Mutation is an important genetic operator which can significantly affect the individual’s development. It marks a minor variation in the genomes by exchanging one component with another. In evolution algorithms, the changes made by mutation might bring substantial differences to chromosomes. For example, a mutation might make a chromosome better in terms of fitness, or the important attributes might be lost due to a random mutation which could result in the decreasing of accuracy and the increasing of processing time.

The critical question is which attribute/terminal should be added or deleted when performing a mutation. Ideally, a weak terminal deleted by the mutation operation should be replaced by a strong one. This can be achieved by using the following improved mutation operation.

**Fig. 4:**
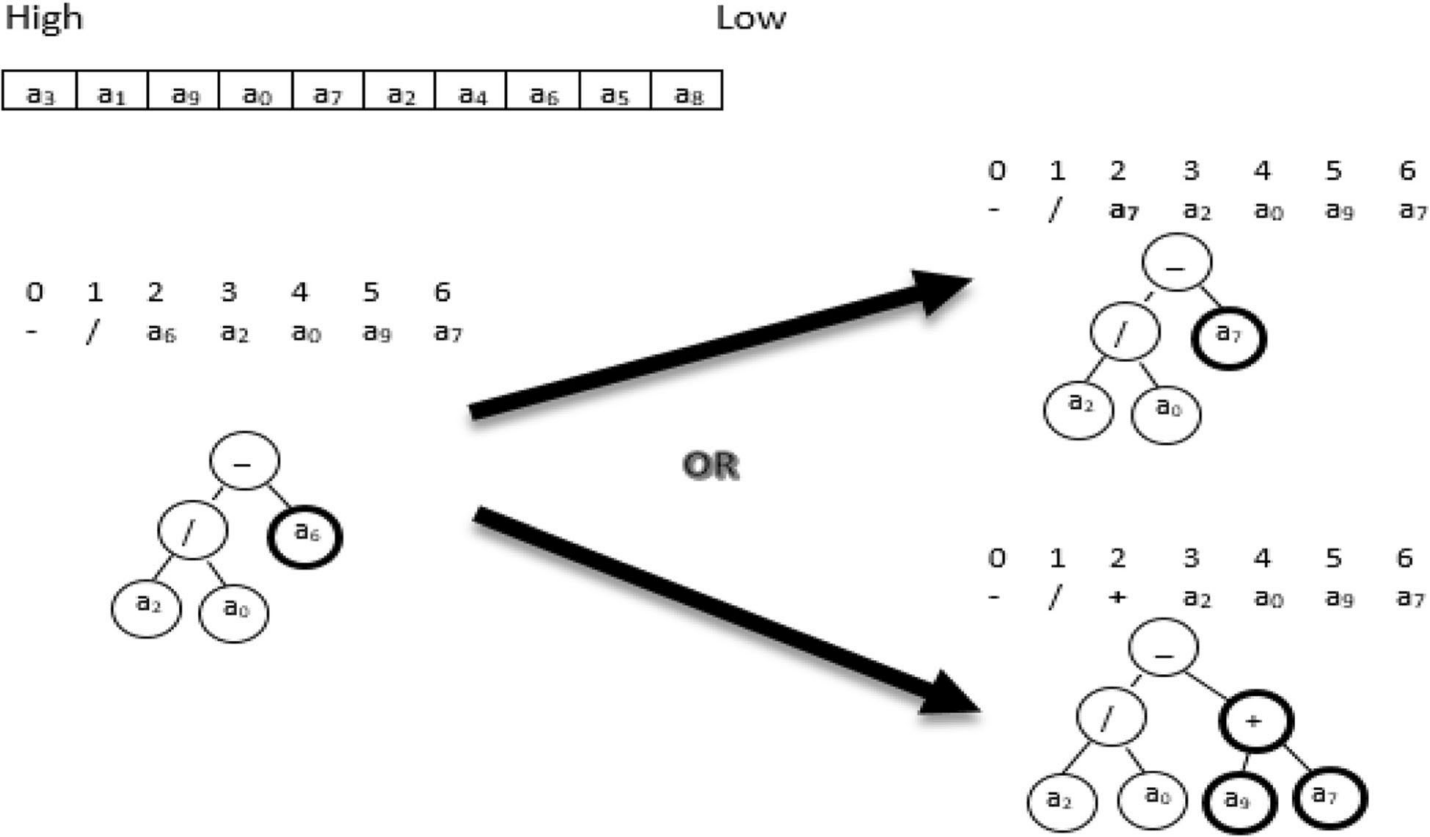
Example of mutation operation for DGS

To clarify the DGS mutation operation, we provide a simple example shown in Fig. 4. In the example, the chromosome consists of a single gene (− / a6 a2 a0 a9 a7). The gene head size (h) is 3. The function set is {Q, +, −, *, /} which means *n* = 2. According to Eq. (2), the gene tail size (t) is 4 and the chromosome length is (3 + 4) =7.

All the terminals in the database are weighed once at the beginning of the program and sorted in a descending order based on their weights as shown at the top of Fig. 4. In this example a_3_ has the highest weight while a_8_ has the lowest weight. Terminal a_6_ is identified by the DGS mutation as the weakest terminal as it has the lowest weight among all terminals in the example chromosome.

For this weak terminal a_6_, DGS mutation has two options to replace it: either it is replaced by a function such as (+) or by a terminal. In the latter option, the replacing terminal should have a weight higher than that of a_6_. In this example terminal a_7_ is selected as a replacing terminal. With the stronger terminals/attributes after mutation, the new chromosome might achieve a higher fitness value than the previous one. The details of this mutation operator are outlined in Algorithm 2.

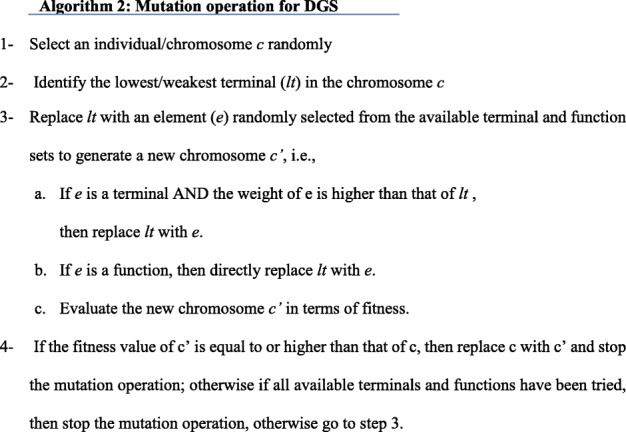



#### Recombination

The second genetic operation we used in this proposed method is the recombination operation.

Generally, in the recombination operation pairs of chromosomes (parents) are randomly selected and combined to generate new pair. To generate the new chromosomes, the parents will exchange one or more parts (short sequences) with each other. The exchanging part can also be the entire gene from one parent with the equivalent gene from the other parent.

**Fig. 5:**
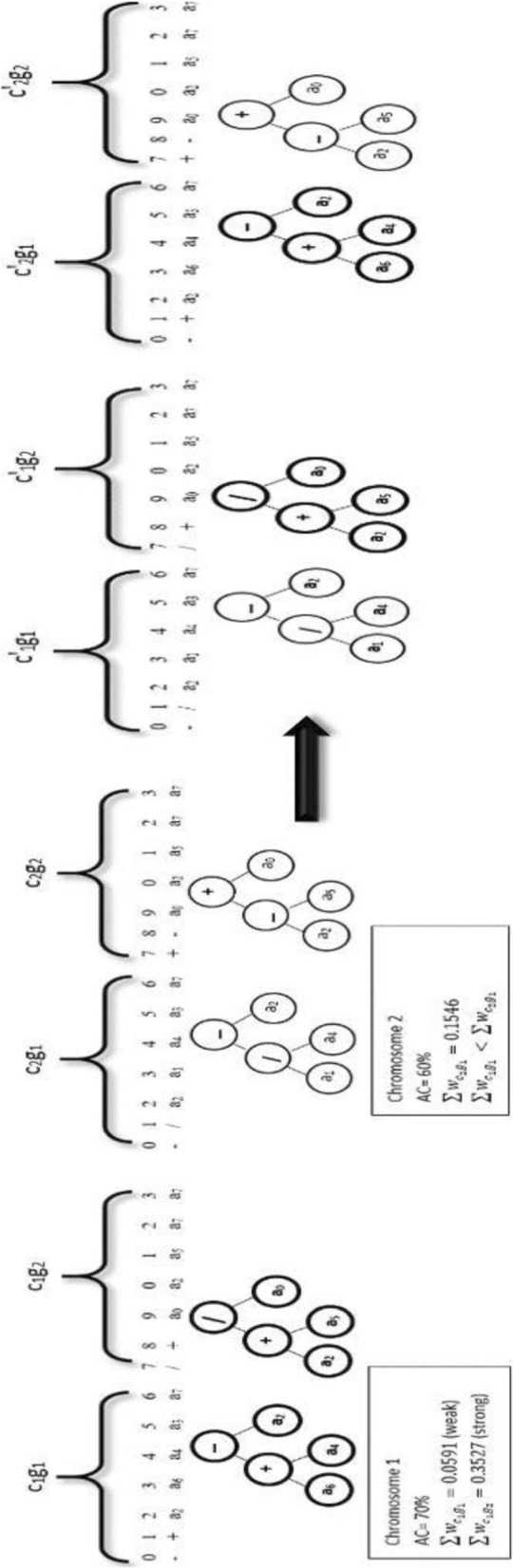
DGS Recombination example

In this study, we replace the random exchange process with a new controlling process. To clarify DGS recombination process we use the example in Fig. 5. DGS program records all the fitness functions for all the chromosomes. The program selects two chromosomes. In this example, the fitness value of chromosome1 is 80% and the fitness value of chromosome2 is 70%. DGS recombination gene operation selects the “strong” gene (gene with the highest weight summation ∑*w*_*i*_) from the chromosome that has a lower fitness value (lc) and exchanges it with the “weak” gene (gene with the lowest weight summation) from another chromosome that has a higher fitness value (hc). The process is repeated until the program obtain a new chromosome (hc’) with a higher fitness value than both parents (the original chromosomes). This idea comes from the gene structure [60].

Based on the above improvements and innovations, the deep gene selectin (DGS) algorithm is presented as pseudocode in Algorithm 3 below.

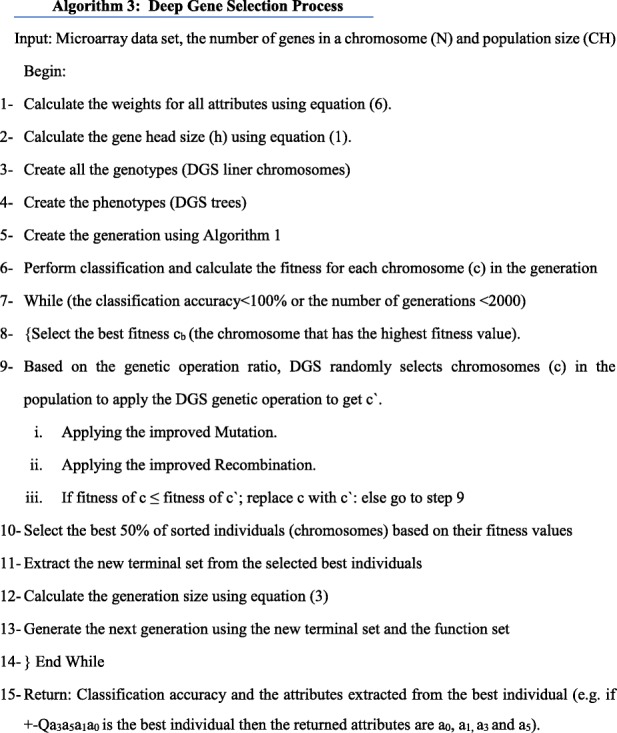



## Data Availability

The lung cancer dataset GSE68465 was downloaded from NCBI.

## Abbreviations

a_0_,----, a_m_: gene coding
AC: Accuracy value
c: Chromosome
CH: the number of Chromosomes in each generation
DGS: Deep Gene Selection
e: element
fs: Functional Set
g: gene
GEP: Gene Expression Programming
GSP: Gene Selection Programming
h: head
hc: higher fitness value
I: the number of iterations
k: the rank value of the attribute
L: Chromosome Length
lt: lowest/weakest terminal in the chromosome
n: the maximum number of parameters needed in the function set
N: the number of genes of a chromosome
r: weight controlling the importance of *AC*
s: the selected number of attributes in the chromosome
t: Tail
T: Terminal size
ts: Terminal Set
w: the weight of each attribute
